# Supplementary material for: Digital interventions for common mental disorders in low- and middle-income countries: A systematic review and meta-analysis
Source: Glob Ment Health (Camb). 2023 Sep 15;10:e68. doi: 10.1017/gmh.2023.50 (PMC10643260; doi:10.1017/gmh.2023.50)
Supplement: Karyotaki et al. supplementary material [file S205442512300050Xsup001.docx]

## PubMed

### Searchstrings:

**# Depression**

"Depression"[Mesh] OR "Depressive Disorder"[Mesh] OR “depress*”[tiab] OR “dysthymi*”[tiab] OR “mood disorder*”[tiab] OR “affective disorder*”[tiab] OR “dysphoric disorder*”[tiab]

**#Anxiety**

“Anxiety disorders” [Mesh] OR "Anxiety"[Mesh] OR "Fear"[Mesh] OR "shyness"[MeSH Terms] OR shyness[tiab] OR shy[tiab] OR “anxiet*”[tiab] OR “agoraphobi*”[tiab] OR “panic”[tiab] OR “social phobi*”[tiab] OR “phobi*”[tiab] OR “obsessive-compulsive”[tiab] OR “neurotic disorder*”[tiab] OR “hoarding*”[tiab] OR “OCD”[tiab] OR “neurotic anxiet*”[tiab] OR “anxious*”[tiab] OR “emotional disorder*”[tiab] OR fear[tiab] OR worry[tiab] OR worrying[tiab] OR worries[tiab] OR GAD[tiab] OR Arachnophobia[tiab] OR Ophidiophobia[tiab] OR Acrophobia[tiab] OR Cynophobia[tiab] OR Claustrophobia[tiab] OR Mysophobia[tiab] OR Aerophobia[tiab] OR Trypophobia[tiab] OR Carcinophobia[tiab] OR Thanatophobia[tiab] OR Glossophobia[tiab] OR Monophobia[tiab] OR Ornithophobia[tiab] OR Alektorophobia[tiab] OR Trypanophobia[tiab] OR Anthropophobia[tiab] OR Aquaphobia[tiab] OR Autophobia[tiab] OR Hemophobia[tiab] OR Xenophobia[tiab] OR Ailurophobia[tiab] OR Nyctophobia[tiab] OR Phobophobia[tiab] OR Philophobia[tiab] OR Triskaidekaphobia[tiab] OR Emetophobia[tiab] OR Entomophobia[tiab] OR Zoophobia[tiab] OR Scelerophobia[tiab] OR Cibophobia[tiab] OR Tokophobia[tiab] OR Pseudodysphagia[tiab] OR Gerascophobia[tiab] OR Technophobia[tiab] OR Ergophobia[tiab] OR Coulrophobia [tiab] OR Photophobia[tiab] OR Numerophobia[tiab] OR Taphophobia[tiab]

**# Psychological interventions**

“Psychotherapy”[Mesh] OR “Counseling”[Mesh] OR psychotherap*[Tiab] OR cbt[Tiab] OR counselling[Tiab] OR counseling[Tiab] OR “Eye Movement Desensitization Reprocessing”[tiab] OR “Eye Movement Desensitization and Reprocessing”[tiab] OR “Eye Movement Desensitisation Reprocessing”[tiab] OR “Eye Movement Desensitisation and Reprocessing”[tiab] OR EMDR[tiab] OR “Bibliotherap*”[tiab] OR mindfulness[Tiab] OR “Autogenic Training”[tiab] OR Logotherap*[tiab] OR "cognitive restructuring"[Tiab] OR "self-control training*"[Tiab] OR "assertiveness training"[Tiab] OR ((“therapy”[SubHeading] OR therap*[Tiab] OR “Therapeutics”[Mesh] OR treatment*[Tiab] OR intervention*[tiab]) AND (“brief psychodynamic”[Tiab] OR “short psychodynamic”[tiab] OR "problem-solving"[Tiab] OR "compassion-focused"[Tiab] OR "compassion-focussed"[Tiab] OR “compassion-based”[tiab] OR constructivist*[Tiab] OR metacognitive[tiab] OR “meta-cognitive”[Tiab] OR "solution-focused"[Tiab] OR "solution-focussed"[Tiab] OR "self-control”[Tiab] OR psychosocial[tiab] OR “peer support“[tiab] OR “task-shifted”[tiab] OR Relaxation[tiab] OR “dialectical behavior”[tiab] OR “emotion-focused”[tiab] OR narrative[tiab] OR “person-centred”[tiab] OR “person-centered”[tiab] OR “Narrative”[tiab] OR “meaning-centered”[tiab] OR “humanistic”[tiab] OR “client-centered”[tiab] OR “meaning-centred”[tiab] OR “client-centred”[tiab] OR “Rogerian”[tiab] OR “Nondirective”[tiab] OR “Non-directive”[tiab] OR “Supportive”[tiab] OR “Life review”[tiab] OR "acceptance and commitment"[Tiab] OR (“schema”[tiab] AND brief[tiab]) OR (“gestalt”[tiab] AND brief[tiab]))) OR "behavior therap*"[Tiab] OR "behaviors therap*"[Tiab] OR "behavioral therap*"[Tiab] OR "behaviour therap*"[Tiab] OR "behaviours therap*"[Tiab] OR "behavioural therap*"[Tiab] OR "cognition therap*"[Tiab] OR “cognitive therap*”[tiab] OR "behavior treatment*"[Tiab] OR "behaviors treatment*"[Tiab] OR "behavioral treatment*"[Tiab] OR "behaviour treatment*"[Tiab] OR "behaviours treatment*"[Tiab] OR "behavioural treatment*"[Tiab] OR "cognition treatment*"[Tiab] OR “cognitive treatment*”[tiab] OR "behavior intervention*"[Tiab] OR "behaviors intervention*"[Tiab] OR "behavioral intervention*"[Tiab] OR "behaviour intervention*"[Tiab] OR "behaviours intervention*"[Tiab] OR "behavioural intervention*"[Tiab] OR "cognition intervention*"[Tiab] OR “cognitive intervention*”[tiab] OR "behavior activation*"[Tiab] OR "behaviors activation*"[Tiab] OR "behavioral activation*"[Tiab] OR "behaviour activation*"[Tiab] OR "behaviours activation*"[Tiab] OR "behavioural activation*"[Tiab] OR exposure[tiab]

**# Internet-based**

"Telemedicine"[Mesh] OR "Mobile Applications"[Mesh] OR "Social Media"[Mesh] OR "Therapy, Computer-Assisted"[Mesh:NoExp] OR "Drug Therapy, Computer-Assisted"[Mesh:NoExp] OR "Telecommunications"[Mesh:NoExp] OR "Electronic Mail"[Mesh] OR "Videoconferencing"[Mesh] OR "Cell Phone"[Mesh] OR "Distance Counseling"[Mesh] OR “Wearable Electronic Devices”[Mesh] OR telehealth[tiab] OR “tele-health”[tiab] OR telepsychology[tiab] OR “tele-psychology”[tiab] OR telepsychiatry[tiab] OR “tele-psychiatry”[tiab] OR “tele-therap*”[tiab] OR teletherap*[tiab] OR “tele-medicine”[tiab] OR telemedicine[tiab] OR telecare[tiab] OR “tele-care”[tiab] OR telecommunicat*[tiab] OR “tele-communicat*”[tiab] OR teleconference*[tiab] OR “tele-conferenc*”[tiab] OR videoconferenc*[tiab] OR “video-conferenc*”[tiab] OR computer*[tiab] OR electronic*[tiab] OR digital*[tiab] OR ehealth[tiab] OR “e-health”[tiab] OR “e-treat*”[tiab] OR “e-therap*”[tiab] OR mhealth[tiab] OR “m-health”[tiab] OR “internet-based*”[tiab] OR “internet treat*”[tiab] OR “internet intervention*”[tiab] OR “internet counsel*”[tiab] OR “distance counsel*”[tiab] OR “web-based*”[tiab] OR cybercounsel*[tiab] OR “cyber-counsel*”[tiab] OR “online treat*”[tiab] OR “online therap*”[tiab] OR “online intervention*”[tiab] OR “online prevention*”[tiab] OR “online counsel*”[tiab] OR “text-messag*”[tiab] OR textmessag*[tiab] OR SMS[tiab] OR texting*[tiab] OR “short message service*”[tiab] OR mobile*[tiab] OR smartphone*[tiab] OR “cell-phone*”[tiab] OR cellphone*[tiab] OR “cellular phone*”[tiab] OR blended*[tiab] OR “software app*”[tiab] OR “handheld device*”[tiab] OR “hand held device*”[tiab] OR iPad*[tiab] OR iPhone*[tiab] OR email*[tiab] OR “e-mail*”[tiab] OR sensor*[tiab] OR wearable*[tiab] OR “social media*”[tiab] OR “social network*”[tiab] OR “e-counsel*”[tiab] OR ecounsel*[tiab] OR palmtop*[tiab] OR telephone*[tiab] OR WhatsApp[tiab] OR Twitter[tiab] OR Facebook[tiab] OR Instagram[tiab] OR forum[tiab] OR chat*[tiab] OR “virtual reality*”[tiab] OR avatar*[tiab] OR “Conversational agent*”[tiab] OR “virtual coach”[tiab] OR “virtual agent*”[tiab] OR “embodied agent*”[tiab] OR “relational agent*”[tiab] OR “interactive agent*”[tiab] OR “virtual character*”[tiab] OR “virtual human*”[tiab] OR “virtual assistant*”[tiab] OR VR[tiab] OR “serious game*”[tiab] OR “serious gaming”[tiab] OR gamification[tiab]

**# Trials/ SR/ Meta-analysis**

"Meta-Analysis" [Publication Type] OR "Meta-Analysis as Topic"[Mesh] OR metaanaly*[tiab] OR meta-analy*[tiab] or metanaly*[tiab] OR "Systematic Review" [Publication Type] OR systematic[sb] OR meta-analysis[Filter] OR systematicreview[Filter] OR "Cochrane Database Syst Rev"[Journal] or prisma[tiab] OR “preferred reporting items”[tiab] OR prospero[tiab] OR ((systemati*[ti] OR umbrella[ti] OR “structured literature”[ti]) AND (review[ti] OR overview[ti])) OR “systematic review”[tiab] OR “umbrella review”[tiab] OR “structured literature review”[tiab] OR “systematic qualitative review”[tiab] OR “systematic quantitative review”[tiab] OR “systematic search and review”[tiab] OR “systematized review”[tiab] OR “systematised review”[tiab] OR “systemic review”[tiab] OR “systematic literature review”[tiab] OR “systematic integrative literature review”[tiab] OR “systematically review”[tiab] OR “scoping literature review”[tiab] OR “scoping review”[tiab] OR “systematic critical review”[tiab] OR “systematic integrative review”[tiab] OR “systematic evidence review”[tiab] OR “systematic integrative literature review”[tiab] OR “systematic mixed studies review”[tiab] OR “systematized literature review”[tiab] OR “systematic overview”[tiab] OR “Systematic narrative review”[tiab] OR “narrative review”[tiab] OR metasynthes*[tiab] OR meta-synthes*[tiab] OR “Randomized Controlled Trial”[pt] OR “Randomized Controlled Trials as Topic”[Mesh] OR “Random allocation” [Mesh] OR “Double-blind method”[Mesh] OR “Single-blind method”[Mesh] OR random[tiab] OR randomly[tiab] OR randomised[tiab] OR randomized[tiab] OR randomising[tiab] OR randomizing [tiab] OR ((singl*[tiab] OR doubl*[tiab] OR trebl*[tiab] OR tripl*[tiab]) AND (mask*[tiab] OR blind*[tiab] OR dumm*[tiab])) OR RCT[tiab] OR "Clinical Trials as Topic"[Mesh] OR “Clinical Trial”[pt] OR “clinical trial*”[tiab] OR “Controlled Clinical Trial”[pt] OR “controlled trial*”[tiab]

**# Publication type**

NOT ("Comment" [Publication Type] OR "Letter" [Publication Type] OR "Editorial" [Publication Type] OR (("Animals"[Mesh] OR "Models, Animal"[Mesh]) NOT "Humans"[Mesh]))
